# Cells with Many Talents: Lymphatic Endothelial Cells in the Brain Meninges

**DOI:** 10.3390/cells10040799

**Published:** 2021-04-02

**Authors:** Irina Suárez, Stefan Schulte-Merker

**Affiliations:** 1 Institute for Cardiovascular Organogenesis and Regeneration, Westfälische Wilhelms-Universität Münster, 48149 Münster, Germany; irinasm@uni-muenster.de; 2 Faculty of Medicine, Westfälische Wilhelms-Universität Münster, 48149 Münster, Germany; 3 Cells-in-Motion Cluster of Excellence, Westfälische Wilhelms-Universität Münster, 48149 Münster, Germany

**Keywords:** lymphatic cells, meninges, endocytosis, regeneration

## Abstract

The lymphatic system serves key functions in maintaining fluid homeostasis, the uptake of dietary fats in the small intestine, and the trafficking of immune cells. Almost all vascularized peripheral tissues and organs contain lymphatic vessels. The brain parenchyma, however, is considered immune privileged and devoid of lymphatic structures. This contrasts with the notion that the brain is metabolically extremely active, produces large amounts of waste and metabolites that need to be cleared, and is especially sensitive to edema formation. Recently, meningeal lymphatic vessels in mammals and zebrafish have been (re-)discovered, but how they contribute to fluid drainage is still not fully understood. Here, we discuss these meningeal vessel systems as well as a newly described cell population in the zebrafish and mouse meninges. These cells, termed brain lymphatic endothelial cells/Fluorescent Granular Perithelial cells/meningeal mural lymphatic endothelial cells in fish, and Leptomeningeal Lymphatic Endothelial Cells in mice, exhibit remarkable features. They have a typical lymphatic endothelial gene expression signature but do not form vessels and rather constitute a meshwork of single cells, covering the brain surface.

## 1. Introduction

Lymphatics have evolved in the vertebrate lineage and are essential for normal development and tissue function to occur. The lymphatic system constitutes a unidirectional, blind-ended vascular network closely associated with the blood vasculature. It has three major functions, the main one being the regulation of fluid homeostasis by taking up the excess of interstitial fluid (ISF) that is not collected by veins. Another important function is the trafficking of immune cells. Lastly, the lymphatic vessels in the small intestine take up dietary fats. The lymphatic capillaries are responsible for the absorption of ISF and exogenous molecules. The cells composing lymphatic capillaries contain discontinuous button-like junctions that allow the entry of fluid, leukocytes, and dendritic cells, among others. The lymph is then conducted to the lymph nodes and lymphoid organs and eventually returned to the general circulation via the lymph ducts. In case of malfunction, ISF accumulates in tissues causing their swelling, a condition known as lymphedema. Most lymphedema cases belong to the group of secondary lymphedema, caused by physical or radiation damage to lymphatic vessels or by associated diseases such as parasitic infections. Primary lymphedemas, in contrast, are caused by genetic mutations in the key molecular players during lymphatic development and are relatively rare. Examples of primary lymphedema are Hennekam syndrome and Milroy disease [1,2,3]. Independent of the cause, lymphedemas are chronic conditions which severely affect the quality of life and for which there is currently no available cure. Lymphatic deficiency has also been linked to chronic inflammation, to metastasis spreading when tumor cells enter the lymphatic vessels, and to adult-onset obesity [2,3,4]. 

While the existence of lymphatic vessels in vertebrates such as humans, rodents, and dogs has long been known, zebrafish lymphatics were only discovered more recently [5,6]. The particular strengths of the zebrafish model, i.e., the ability to carry out forward genetic screens with the aim to identify novel gene functions involved in lymphatic development, and the ability to perform in vivo imaging, has helped to make a number of key contributions to the lymphatic field, some of which will be discussed here. 

Lymphatic vessels are present in all organs and tissues except for few where, presumably because of small quantities of ISF or the existence of alternative draining systems, lymphatics have not been reported. These include bone, bone marrow, and adipose tissue, among others [7], but the most notable of all is arguably the brain. With its high energetic demand and metabolic turn-over, and the potentially fatal consequences of brain edema, one would assume that the brain contains a vast and well-developed lymphatic network. Conversely, brain parenchyma is devoid of lymphatic vessels. Hence, alternative mechanisms must exist that substitute functionally for the absence of lymphatic vessels within the brain. This review will focus on the advances that have been achieved in the study of macromolecule clearance from the brain, especially in zebrafish.

## 2. Lymphangiogenesis

Lymphangiogenesis is a highly dynamic process that commences at E9.5 in mice and at 32 h post fertilization (hpf) in zebrafish. In both model organisms, many cellular events and most of the molecular players driving lymphangiogenesis are highly conserved. The development of lymphatic structures starts with the up-regulation of the lymphatic marker Prospero homeobox protein 1 (PROX1) in a subset of venous endothelial cells. In mice, these cells are located within the dorsal side of the cardinal vein and in zebrafish within the posterior cardinal vein (PCV) from where they sprout dorsally [8,9,10], eventually giving rise to the main lymphatic vessels. This process is exquisitely dependent on the Vascular endothelial growth factor C (VEGFC) protein and its receptor Vascular endothelial growth factor receptor 3 (VEGFR3). VEGFC needs to be proteolytically processed at both ends in order to become fully biologically active. This processing is accomplished by FURIN/PC5 at the C-terminus and A disintegrin and metalloprotease with thrombospondin motifs 3 (ADAMTS3) at the N-terminus. ADAMTS3 is recruited by the Collagen and calcium binding EGF domains 1 (CCBE1) protein. Mice deficient for either of these key molecules display a complete lack of lymphatic structures in those cases where mutants can be analyzed [11,12,13,14,15,16,17]. While in zebrafish the same applies for *vegfc* and *ccbe1* [5,6,18], only double mutants for *adamts3* and *adamts14* show lymphatic defects, as both proteases have a redundant role during lymphangiogenesis. Interestingly, also human ADAMTS14 has been shown to process VEGFC in vitro, suggesting that its VEGFC-processing function might be conserved in mammals [19]. Lymphangiogenesis as such and other molecular players involved in this process have been previously reviewed in great detail for both zebrafish [20,21] and mice [4,22,23].

It has been traditionally believed that lymphatic endothelial cells (LECs) arise exclusively from venous structures. This view has been challenged over the last few years based on the discovery of LECs from non-venous origins in different mouse organs, such as the heart, the dermis, and the mesentery [24,25,26,27]. In zebrafish, an equivalent example is found in the case of facial lymphatics, which differ from trunk lymphatics in that their formation relies on three different cellular sources, one of them non-venous [28,29]. Nevertheless, the development of the facial lymphatic system also depends on the Vegfc signaling pathway [28].

## 3. Fluid and Macromolecule Clearance from the Brain

For many years, the brain and the meninges were considered devoid of lymphatic structures. Because of its high metabolic activity, the brain produces and turns over large quantities of macromolecules and waste products that need to be cleared to maintain proper physiological functionality. Many efforts have been undertaken in order to explore the alternative mechanisms that the brain must have developed in order to remove waste products and to maintain fluid homeostasis, and the cerebrospinal fluid (CSF) was proposed as a ‘waste sink’ [30] almost a century ago. To further understand how the CSF could perform this function, injections of tracer molecules into the brain parenchyma and into the CSF in the subarachnoid space were carried out [31]. This study argued that the CSF is able to leave the subarachnoid space and travel along perivascular spaces into deep brain areas, and it is then cleared also through perivascular spaces. In addition, the ISF uses these same routes to clear macromolecules from the parenchyma. It has been proposed that the efflux of fluid from the brain parenchyma towards the perivascular space occurs via bulk flow, i.e., the combined movement of water and solutes following a pressure gradient [32,33]. The topic has raised interest again in the last decade after Iliff and colleagues shed new light on this mechanism aided by the use of advanced fluorescence microscopy [34]. They proposed a clearance mechanism for molecules in the brain that includes movement of CSF from the subarachnoid space into peri-arterial spaces, subsequent entry into the brain parenchyma where it mixes with the ISF, and a common efflux route via peri-venous spaces [34]. In addition to the characterization of peri-arterial and peri-venous spaces as transport routes for fluids, the authors suggest a critical role for the glia to ensure proper functioning of the system and consequentially coined the term “glymphatic system”. Within the central nervous system (CNS) endothelial capillaries are surrounded by astrocytic end-foot processes and the glia limitans [35], and it is the space between the basement membrane of blood vessels and the glia limitans that is defined as the perivascular space. The astrocytic end-feet express the water channel aquaporin 4 (AQP4) [36]. Iliff et al. showed that at the level of the arterioles, water and solutes in the CSF use AQP4 to reach the interstitium, whereas bigger molecules have to pass through spaces between the astrocyte end-feet. Interestingly, AQP4 null mice exhibited significantly reduced clearance levels of molecules from the interstitium, suggesting that fluid passing through the astroglial AQP4 channel reinforces the bulk flow needed to clear macromolecules from the interstitium. This idea was supported by the observation of higher levels of AQP4 expression in venules compared to arterioles, thereby helping to potentially keep a low resistance path for the water and waste to drain from the interstitium into peri-venous spaces [34]. A well-known ISF solute in the brain is Amyloid β, whose accumulation in the parenchyma is a risk factor for Alzheimer’s disease [35]. AQP4 null mice show significant reduced Amyloid β clearance from the interstitium [34], suggesting that the glymphatic system could be involved in the progression of age-related neurodegenerative diseases. Upon ISF drainage into the perivascular space of parenchymal venules, the ISF exits mainly via two venous routes: the medial internal cerebral veins and the lateral–ventral caudal rhinal veins [34].

The CSF was thought to mainly drain into the blood system via the arachnoid granulations until a series of publications evidenced that part of the CSF-ISF exited along the olfactory nerve to enter the nasal lymphatics and ending in cervical lymph nodes [37]. Recently, a network of lymphatic vessels was discovered in the dura mater of mice, but direct routes from the brain parenchyma to the dura have not been described to date [38,39]. These vessels are also present in humans and non-human primates [40], and just like peripheral lymphatics, meningeal lymphatics are implicated in immune cell trafficking [38]. Different studies have linked impairment of both the glymphatic system and the meningeal lymphatics function to diverse neurodegenerative diseases and brain injuries [40,41,42], and this is naturally a topic of considerable excitement.

In addition to the modes of CSF-ISF clearance described above, other routes for fluid drainage from the brain were described and reviewed elsewhere [42,43,44,45,46]. Currently, this is still a controversial field and some findings are, at least at first sight, contradictory [42,44]. Technical issues have to be overcome, such as alteration of fluid pressures upon tracer injections, duration of experiments, and anesthesia regimes, in order to standardize experimental set-ups. There is evidence for an increase of CSF outflow in awake mice compared to anesthetized mice [47] as well as of a differential effect of different anesthetic conditions in the glymphatic system activity [48].

**Figure 1:**
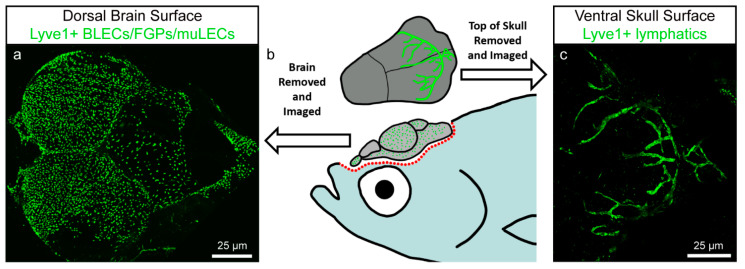
Zebrafish brain lymphatic endothelial cells (BLECs)/Fluorescent Granular Perithelial cells (FGPs)/mural lymphatic endothelial cells (muLECs) and meningeal lymphatic vessels in adult specimens. Image modified from Castranova et al. [49]. (**a**) Dorsal confocal image showing BLECs/FGPs/muLECs extended over the whole brain surface. (**b**) Schematic representation of an adult zebrafish depicting the dissection procedure for imaging of BLECs/FGPs/muLECs and intracranial meningeal lymphatic vessels. (**c**) Confocal image of the inner surface of the skull showing the meningeal lymphatic vessels that stay attached to the skull after dissection.

A very recent study has revealed the presence of a similar network of intracranial lymphatic vessels covering the inner lining of the zebrafish skull (Figure 1) [49]. These vessels, comparable to mice meningeal lymphatics, are capable of draining substances from the brain and transporting immune cells. The origin of the zebrafish meningeal vessels are cells that sprout from the facial lymphatics in 9 dpf specimens and that migrate rostrally and laterally first, eventually spanning large brain areas, especially in the region of the cerebellum and the optic tecta. The connection to the facial lymphatics is eventually lost and further studies need to be done to properly understand where they exactly drain their content. The network is fully developed in 1.5-month-old fish, although vascular complexity seems to increase with age. The growth of these vessels is stimulated by the administration of VEGFC, an observation also reported in the case of mammalian meningeal lymphatics [49].

## 4. BLECs/FGPs/muLECs

**Figure 2:**
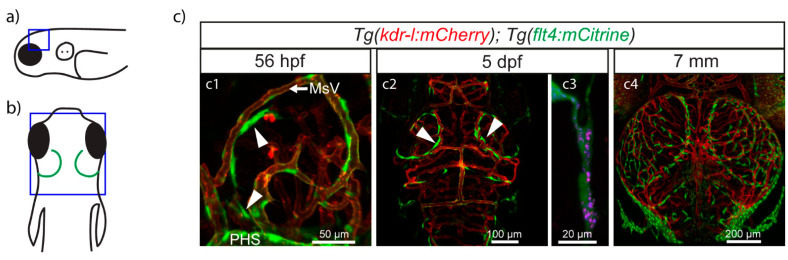
Zebrafish BLECs in embryonic and larval specimens. (**a**,**b**) Schematic representations of zebrafish showing the same orientation and close-up regions (blue squares) as in c1 (**a**) and c2-c4 (**b**). (**c**) Confocal images from van Lessen et al. [50]. Lympho-venous structures are depicted in green and blood vessels in red. BLECs (arrow heads) start sprouting at 56 hpf from behind the PHS and migrate along the MsV (**c1**). By 5 dpf they have formed a bilateral loop in the TeO (**c2**). Cells in the loop are able to take up injected dyes such as pHrodo (red) and IgG-Alexa647 (blue) (**c3**). BLECs keep spreading above the whole brain area (**c4**), where they will stay as single cells throughout the lifespan of the animal. Dpf, days post fertilization; hpf, hours post fertilization; MsV, mesencephalic vein; PHS, primary head sinus; TeO, optic tectum.

In 2017, previous to the description of meningeal lymphatic vessels in zebrafish, three different groups reported the presence of isolated cells expressing lymphatic markers in the zebrafish meninges. They each termed these cells differently, either brain lymphatic endothelial cells (BLECs) [50], Fluorescent Granular Perithelial cells (FGPs) [51], or mural lymphatic endothelial cells (muLECs) [52]. For simplicity reasons, we will refer to these cells as BLECs. This unique subpopulation of cells stays attached to the surface of the brain (Figure 1a,b) when dissecting the specimen, as opposed to the meningeal lymphatic vessels, which adhere to the inner surface of the skull (Figure 1b,c). BLECs reside in close proximity with meningeal blood vessels without sharing a common basement membrane and contain electron dense cytoplasmic inclusions resembling endocytic vesicles. The use of transmitted electron microscopy and double transgenic lines allowed to rule out the possibility that these new cells have a pericyte, macrophage, microglial, glial, neutrophil, smooth muscle, oligodendrocyte, or blood endothelial cell identity. Instead, these cells express well-known endothelial lymphatic markers such as *vegfr3*, *prox1a*, the *hyaluronic acid receptor 1* (*lyve1),* as well as the pan-endothelial marker *friend leukemia integrated 1a* (*fli1a*) and the *mannose receptor C type 1a* (*mrc1a*), which is also expressed in macrophages. RNAseq expression profiling confirmed lymphatic endothelial identity [51,52]. Consistent with their lymphatic nature, the development of BLECs depends on the Vegfc/Vegfr3 signaling pathway [50,52]. Similar to the trunk lymphatic vasculature, BLECs originate from a venous structure: they sprout from the choroidal vascular plexus (CVP) near the primary head sinus (PHS) at 56 hpf and migrate along the mesencephalic vein (MsV) (Figure 2(a,c1)). At 5 dpf, they form a bilateral loop positioned over the optic tectum (TeO) (Figure 2(b,c2)) and cease to be motile while acquiring a more mesenchymal morphology [50,51,52]. During further development, the BLECs will increase in number to populate almost the whole surface of the brain (Figure 1a; Figure 2(b,c4)), but they will remain within the meningeal layer without entering the brain parenchyma [50,51,52]. This accumulation of cells over time is due to recruitment of new cells, since BLECs as such do not have a high proliferative activity [51]. Strikingly, unlike other LECs, BLECs do not ultimately form lymphatic vessels but stay as loose single cells [50,51,52]. Despite covering most of the surface of the brain, differences were noted in their density and morphology across different brain areas, e.g., tectal BLECs are densely packed and have long processes, whereas cerebellar BLECs are sparse, thin, and elongated [50]. The common denominators for these cells, independent of their anatomical location, are the expression of lymphatic markers and the presence of large cytoplasmic vesicles. With the current knowledge on the variety of lymphatic markers expressed by BLECs, their venous origin, and their dependence on the Vegfc/Vegfr3 signaling pathway for sprouting and migration, and in the absence of evidence for non-lymphatic cells contributing to this cell population, it has been accepted that these cells are of lymphatic nature. 

BLECs have shown, in different studies, a number of interesting features and exhibit some remarkable characteristics and behaviors. In the following, we will discuss each of these features separately.

Particularly the presence of intracellular inclusions provided an indication of a potential clearance role for these cells. In a series of intracerebral and intraventricular injections using polysaccharides and glycoproteins of different sizes, BLECs were shown to take up macromolecules ranging from 10 kDa to at least 150 kDa [50]. When injected elsewhere (intramuscularly), BLECs were not able to internalize Q-dots [51]. Co-injection of a pH sensitive dye (pHrodo) demonstrated that macromolecules indeed end up in acidic vesicles in the cytoplasm of BLECs (Figure 2(c3)). Since this process is blocked by inhibition of dynamin, BLECs apparently internalize cargo via endocytosis [50]. A possible key player in the endocytosis process is Mrc1a, which is expressed by BLECs and has been shown to be involved in uptake-processes in mammals [53]. Injecting the mannose receptor inhibitor mannan blocked the internalization ofIgG-Alexa647 [50].

Given their relevance for human brain pathology, special interest focused on Amyloid β and Tau. Injection of Amyloid β into the CSF of larval zebrafish [54,55] resulted in its internalization by BLECs. Microglia have long been established as the main cells removing extracellular debris from the brain [56]; hence, identifying a cell population with high endocytic capacity immediately adjacent to the brain raises the question about ‘division of labor’ between microglia and BLECs, particularly as BLECs appear to be more efficient in direct comparison [54]. A formal analysis is required in order to decipher the differences between BLECs and microglia in terms of substrate specificity and context of action.

The notion of endothelial cells that do not line classical vessels and that are able to take up substances from the immediate environment is reminiscent of scavenger endothelial cells (SECs). SECs are non-macrophagic cells with high endocytic capacity, specialized in scavenging macromolecules and nanoparticles present in the blood [57,58] and, in mice models, also blood-borne viruses [58]. In mammals, SECs are located in the liver (LSECs), but in fish, they have been found in different organs: kidney, heart, or gills [58]. Recently, Campbell et al. showed the presence of SECs in several zebrafish veins, including the embryonic cardinal vein, and that their clearance mechanism is highly dependent on the transmembrane receptor stabilin-2 (Stab2) [57]. Comparing embryonic SECs within the PCV and larval BLECs within the meninges in terms of scavenging exogenous substances led to the conclusions that BLECs qualify as SECs [54].

A second physiological role that BLECs appear to fulfil is their interaction with blood vessels. The physical proximity of BLECs, which are commonly found immediately juxtaposed to blood vessels (Figure 2), indeed suggests some form of interdependence. This is supported by RNAseq data [51,52] revealing BLECs to express pro-angiogenic factors such as *vegfaa* and *vegfab*, provoking the question whether they play a role during angiogenesis. Using *vegfc; vegfd* double heterozygous fish, Bower et al. noted that embryos with fewer BLECs and displaying an asymmetrical distribution of these cells between the brain hemispheres also displayed the same asymmetry of blood endothelial cells (BECs) in the meningeal blood vasculature. Transheterozygous individuals did not, however, show meningeal blood vessel leakage. The interdependence hypothesis is reinforced by observations made when carrying out ablations of BLECs in just one hemisphere, which result in a reduced number of BECs in that hemisphere compared to the contralateral one [52]. Together, these results suggest BLECs to play a role in developmental angiogenesis of the meningeal blood vasculature.

In yet another and more recent study, Chen et al. unveiled regenerative functions of BLECs during pathological conditions in the case of brain vascular injury [59]. Using an inducible version of the NTR/MTZ system [60], massive damage of the vasculature of the brain parenchyma was inferred [59]. Two days after the injury, new blood vessels started to form from adjacent areas, for a period of seven or eight days after the injury. Surprisingly, two days after inducing the injury, lymphatic cells were apparent in the traumatized parenchymal area, correlating with the onset of blood vascular regeneration. These ingrowing cells express *lyve1*, *fli1a*, and *vegfr3*, confirming LEC identity. Moreover, lineage tracing experiments showed that BLECs contribute to the ingrown LECs [59], an interesting fact given that it was previously observed that BLECs are neither proliferative nor mobile, other than during their growth phase [51]. The ingression of LECs into the brain parenchyma is dependent on Vegfc, which is expressed by remaining BECs and some neurons in the injured area [59]. Because BLECs also express *vegfc*, it was hypothesized Vegfc may function as a pro-lymphangiogenic signal in adult meninges [52]. It is plausible that the aforementioned expression of pro-lymphangiogenic factors contributes to the signaling from BECs to ensure fast and proper LEC migration. Notably, and unlike for BLECs under normal conditions, the ingrowing LECs form lumenized lymphatic vessels that possess the ability to drain ISF. LECs migrate as single cells to the injured area and start forming lymphatic vessels between days 2 and 3 after the injury, with vessels becoming fully functional at day 3. In a similar process, LECs in murine skin regeneration models migrate as singular cells in the direction of the ISF flow and start organizing vessels [61]. Accordingly, and corroborating the importance of *vegfc* for the process, in *ccbe1* mutant fish, LECs do not enter the parenchyma, the blood vasculature fails to regenerate, and injured fish die from cerebral edema. Along with their task in resolving the edema resulting from the vascular injury, and in contrast to developmental lymphangiogenesis, the newly formed lymphatic vessels serve as migrating routes for the regeneration of the blood vasculature. The mechanism through which lymphatic vessels guide the growth of blood vessels is still not fully understood, but an increase in the expression of surface adhesion molecules between both vessel types was observed, suggesting physical contact [59]. Once blood vessels are restored, lymphatic vessels become apoptotic and will eventually disappear, thereby reinstating a situation where the brain parenchyma is again devoid of lymphatic vessels. Similar results were also reported in a photochemical thrombosis model, which mimics human cerebral thrombosis [59]. This requires further work, as other studies in laser-ruptured blood vessel injuries report on macrophages resolving thrombi, instead of ingrown lymphatics [62]. Chen et al. hypothesized that one possible explanation for the different course of action would be that thrombosis and ischemic stroke induce parenchymal edema, whereas vessel rupture constitutes a localized damage. Accordingly, Galanternik et al. described that after injuring meningeal blood vessels lined with BLECs with a laser, neutrophils appeared in the area to repair the damage while BLECs remained immobile [51].

Transient clusters of isolated LECs were described previously, e.g., in the lung, heart, mesentery, and skin of mice. These loose LECs eventually coalesce to form lymphatic vessels or capillaries [25,27,63,64]. Likewise, the zebrafish heart also contains clusters of isolated LECs throughout the life of the animal which will contribute to the formation of capillaries [63]. However, in contrast to these previously described LECs, BLECs constitute a unique LEC population as they retain single cell status throughout the life of the animal and will only contribute to the formation of lumenized lymphatic vessels under certain pathological circumstances, such as injuries causing brain parenchymal edema.

## 5. Meningeal Lymphatic Cells in Mammals

**Figure 3:**
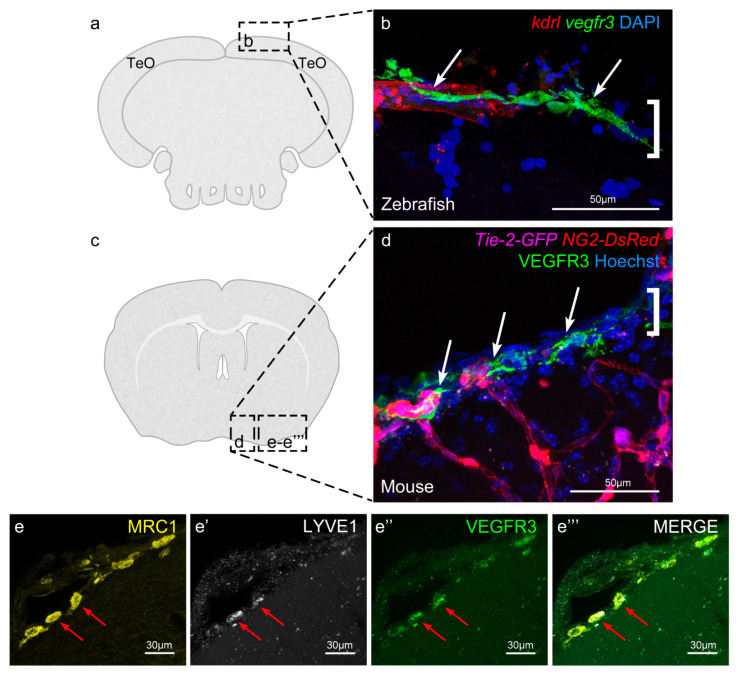
Murine leptomeninges contain cells expressing BLEC markers. Image adapted from Shibata et al. [55]. (**a**) and (**c**) Coronal sections of a zebrafish and a mouse brain, respectively, equivalent to the imaging areas in (**b**,**d**). To facilitate comparison, (**b**,**d**) are both displayed with the parenchyma at the bottom and the meninges at the top. (**b**) Zebrafish BLECs (white arrows) express Vegfr3 (green) and are located adjacent to a meningeal blood vessel (red). The area of the meninges is marked with a white bracket. DAPI (blue) highlights the nuclei. (**c**) IHC of a 17 week old mouse reveals the presence of VEGFR3 expressing cells (white arrows) adjacent to meningeal blood vessels (magenta). These cells are limited to the region of the meninges (white bracket) and do not penetrate into the brain parenchyma. NG2 (red) labels pericytes and smooth muscle cells, Hoechst (blue) marks nuclei. (**e**–**e’’’**) IHC of mouse brain section showing that the cells within the meningeal layers express the BLEC markers MRC1, LYVE1 and VEGFR3.

The discovery of LECs covering the surface of the zebrafish brain raised interest in whether this is a conserved feature in other vertebrates, especially in mammals. Using confocal imaging, Shibata et al. have recently reported the presence of cells in the leptomeninges (i.e., arachnoid mater and pia mater) of mice and humans containing large, homogeneous, electron-dense inclusions (Figure 3a–d) [55]. As in zebrafish, the newly discovered murine leptomeningeal cells reside near blood vessels and express the lymphatic markers VEGFR3, PROX1, and LYVE1, together with MRC1 (Figure 3e–e’’’). For this reason, they have been named Leptomeningeal Lymphatic Endothelial Cells (LLECs) [55]. Antibody staining and immunogold electron microscopy experiments ruled out a pericyte, smooth muscle cell, or macrophage identity. Supporting the latter and mirroring results obtained in zebrafish [50,51], LLEC development was not disturbed in PU.1 knockout mice, which lack myeloid-derived cells. When scrutinized for internalization capacity, LLECs were able to internalize 10 kDa blue dextran and Amyloid β [55]. The question of LLEC origin and development requires further elucidation.

In 1979, Mato and colleagues described a cell type containing electron-dense inclusions residing in the perivascular space of rat brain blood vessels. A very distinguishable feature of these cells is the autofluorescence that their intracellular inclusions emit and that has earned these cells their name: fluorescent granular perithelial cells (FGPs)/Mato cells [65]. The inclusions in Mato cells are of homogenous size in young animals but get heterogeneous with age [66] and were characterized as lysosomes in which the molecules taken up by the cells accumulate. These molecules include lipids from the blood stream and other endogenous and exogenous substances from the CSF [67,68]. As a consequence of their internalization capacity, Mato cells are reminiscent of brain scavenger cells, and based on their morphology, size, and localization, they were classified as distinct from pericytes, astrocytes, and microglia. However, due to the lack of proper markers, their cell identity remained initially unknown, but subsequent immunostaining experiments showed that Mato cells express scavenger receptors and macrophage markers [68]. Correspondingly, more recent studies described the presence of FGPs in the human brain [68] and retina [69] as well as in the mouse retina, where immunocytochemistry was used to show the expression of typical macrophage markers within them [69]. In some studies, auto-fluorescence was not detected, neither in the newly described cells in murine leptomeninges, nor in zebrafish BLECs [55]. This is in contrast with a previous experiment in which auto-fluorescence was reported in zebrafish BLECs/FGPs, causing the authors to propose these cells as equivalent to mammalian FGPs [51]. While this difference could indicate different cell types, another plausible explanation for the incongruous results might be a difference in the diet, namely in the content of fat [55]. To further complicate matters, cells distinct from BLECs and with similar inclusions to FGPs (i.e., smaller and more heterogeneous) were evidenced in transmission electron microscopy images of the zebrafish brain [50]. Despite the similarities which BLECs, LLECs and Mato cells share when it comes to the presence of intracellular vesicles and their scavenging function, a very striking difference is their anatomical distribution. While BLECs and LLECs are only present in the meninges of zebrafish and mice, respectively [50,51,52,55], Mato cells are located in the Virchow–Robin space of arterioles and venules, penetrating into the cortex and spreading throughout all areas of the gray matter [68,70]. A summary of the main characteristics of BLECs, LLECs, Mato cells, and meningeal lymphatic vessels is provided in Table 1. Additional work is required before it is possible to conclude whether they constitute the same cell type. A better description of markers expressed by Mato cells as well as a direct comparison of Mato cells and LLECs on the functional level is required before a definitive statement on this issue can be made.

The discovery of LLECs in rodents and humans adds additional levels of complexity to the issue of macromolecular clearance and tissue homeostasis within the mammalian brain. The co-existence of possibly four mechanisms, exerted by LLECs, the dural lymphatic vessel network, the glymphatic system, and the efflux route along nerves, affirms the complexity of cell types and mechanisms required to ensure proper brain function/physiology. However, how these different systems coordinate is not yet understood. More work is required to fully characterize how each of the systems works separately and how they complement each other, both in a physiological and in a pathological setting. There are studies that show the detrimental effect of age on meningeal lymphatic vessels [41,75], the functionality of CSF efflux [76], and the glymphatic system [77], which as a result can contribute to or lead to neurodegenerative diseases. It is possible that LLECs (and BLECs in zebrafish) lose their physiological ability with age as well. Other important questions are if and how LLECs are involved in the resolution of brain damage and parenchymal edema. Given that BLECs/LLECs are lymphatic cells immediately juxtaposed to the CNS, one would intuitively assume that they are the ones to solve the damage, as has been reported in zebrafish [52,59]. Of note, during zebrafish cryoinjury-induced heart regeneration, isolated LECs are the first to mobilize towards the injury site, and only at later time points, ventricular lymphatic vessels do the same [63,78].

## 6. Conclusions

In recent years, the presence of lymphatic cells residing within the meninges has become evident in both zebrafish and mice. Specifically, BLECs/FGPs/muLECs constitute a unique lymphatic cell population due to their characteristic single cell nature. They exhibit unusual functional properties and a remarkable capacity of contributing to brain vascular regeneration. Moreover, they also display a high efficiency in the uptake of a large variety of macromolecules. There is very good evidence that LLECs might represent the mammalian equivalent of these cells [55]. How these cells coordinate their function in concert with ‘classical’ lymphatic vessels in the meninges of zebrafish and mice is an exciting scientific avenue for the immediate future and requires developing more functional models which also allow physiological properties of the brain to be tested.

How LLECs in mice relate to Mato cells/FGPs in mice warrants further studies. The phagocytic properties are common to both cell types, but there are differences. One difference, concerning diverging reports on auto-fluorescence of lysosomal content, might well be technical and depend on diet [55]. Furthermore, differences on the type of lysosomal appearance were reported (foamy in Mato cells, homogeneous in LLECs), but also here one could argue about age dependence or other physiological matters. A third distinguishing feature, however, between LLECs and Mato cells concerns anatomical location: LLECs have been exclusively reported associated with the meninges, while Mato cells have been documented in the cortex, most often associated with arterioles, but not capillaries [68,70]. Hence, at present, one has to at least consider the possibility that LLECs and Mato cells are distinct cell populations. Lineage studies might have to be employed to resolve this issue.

The study of lymphatics associated with the brain is comparatively novel, and some of the work, especially in zebrafish, is very recent. Considerable work is yet to be done to fully understand the system and get a complete appreciation of how the brain is cleared from macromolecules and fluids both in physiological and pathological conditions. The transparency during embryogenesis and the relative easiness to generate transgenic lines have made zebrafish an ideal model organism for the study of lymphangiogenesis and related dynamic processes. Together with the recent discoveries of potentially equivalent lymphatic structures in mice and zebrafish meninges, zebrafish will undoubtedly remain an advantageous model system for the investigation of brain lymphatics.

## Figures and Tables

**Table 1:** Summary of various characteristics of zebrafish BLECs, mammalian Leptomeningeal Lymphatic Endothelial Cells (LLECs), mammalian Mato cells, and zebrafish and mammalian meningeal lymphatic vessels.

	BLECs/FGPs/muLECs	LLECs	Mato Cells	Meningeal Lymphatic Vessels
Organism	Zebrafish [50,51,52]	Mammals [55]	Mammals [65]	Zebrafish and mammals [38,39,49]
Localization	Meninges, outside basement membrane of meningeal blood vessels [50,51,52]	Leptomeninges, outside basement membrane of meningeal blood vessels [55]	Brain cortex, in the Virchow-Robin space of arterioles and venules [68,70]	Dura mater in mice, meninges in zebrafish [38,39,49]
Marker/gene expression	Lymphatic marker genes (*vegfr3, prox1, fli1a, stabilin 1 and 2, mafba, lyve1b nrp2a); mrc1a*; pro-lymphangiogenic and angiogenic factors [50,51,52]	Lymphatic marker genes (Prox1, Lyve1, Vegfr3); Mrc1 [55]	Macrophage marker genes (1a antigen, Fcγ2a and 2b) Type I and II Scavenger Receptors [68,71]	Lymphatic marker genes (*lyve1b* and *prox1* in zebrafish, Lyve1, Prox1, Vegfr3, Podoplanin in mice); *mrc1a* in zebrafish, CD31 in mice [38,39,49]
Function	Uptake of exogenous substances; blood vessel regeneration in trauma model [50,51,52,59]	Uptake of Amyloid β and Dextran [55]	Scavenging function [66,68,69,72]	Fluid drainage from the brain, transport of immune cells [38,39,49]
Prominent lysosomal vesicles	Large, circular, homogeneous [50,51,52]	Large, circular, homogeneous [55]	Size and shape variable depending on age and diet [73]	None
Origin	Venous choroidal vascular plexus. Not of haemopoietic origin [50,51,52]	Unknown, but not of haemopoietic origin [55]	Unknown, but suggested to derive from leptomeningeal cells [74]	Facial lymphatics in fish, at least in part [49]. Unclear in mammals.

## Data Availability

No new data were created or analyzed in this study. Data sharing is not applicable to this article.
